# Evaluation of commercially available point-of-care ultrasound for automated optic nerve sheath measurement

**DOI:** 10.1186/s13089-023-00331-8

**Published:** 2023-08-02

**Authors:** Brad T. Moore, Tom Osika, Steven Satterly, Shreyansh Shah, Tim Thirion, Spencer Hampton, Stephen Aylward, Sean Montgomery

**Affiliations:** 1 Medical Computing, Kitware, Inc, Carrboro, NC USA; 2 Surgical Critical Care, Duke University Health System, Durham, NC USA; 3 Trauma, Acute, and Critical Care Surgery, Duke University Health System, Durham, NC USA; 4 Duke University Health System, Durham, NC USA; 5 Neurocritical Care, Duke University Health System, Durham, NC USA

**Keywords:** Optic nerve sheath, Ocular ultrasound, Ultrasound, Intracranial pressure, Traumatic brain injury, Point-of-care ultrasound, Image analysis, Ultrasound quality assurance

## Abstract

**Background:**

Measurement of the optic nerve sheath diameter (ONSD) via ultrasonography has been proposed as a non-invasive metric of intracranial pressure that may be employed during in-field patient triage. However, first responders are not typically trained to conduct sonographic exams and/or do not have access to an expensive ultrasound device. Therefore, for successful deployment of ONSD measurement in-field, we believe that first responders must have access to low-cost, portable ultrasound and be assisted by artificial intelligence (AI) systems that can automatically interpret the optic nerve sheath ultrasound scan.

We examine the suitability of five commercially available, low-cost, portable ultrasound devices that can be combined with future artificial intelligence algorithms to reduce the training required for and cost of in-field optic nerve sheath diameter measurement. This paper is focused on the quality of the images generated by these low-cost probes. We report results of a clinician preference survey and compare with a lab analysis of three quantitative image quality metrics across devices. We also examine the suitability of the devices in a hypothetical far-forward deployment using operators unskilled in ultrasound, with the assumption of a future onboard AI video interpreter.

**Results:**

We find statistically significant differences in clinician ranking of the devices in the following categories: “Image Quality”, “Ease of Acquisition”, “Software”, and “Overall ONSD”. We show differences in signal-to-noise ratio, generalized contrast-to-noise ratio, point-spread function across the devices. These differences in image quality result in a statistically significant difference in manual ONSD measurement. Finally, we show that sufficiently wide transducers can capture the optic nerve sheath during blind (no visible B-mode) scans performed by operators unskilled in sonography.

**Conclusions:**

Ultrasound of the optic nerve sheath has the potential to be a convenient, non-invasive, point-of-injury or triage measure for elevated intracranial pressure in cases of traumatic brain injury. When transducer width is sufficient, briefly trained operators may obtain video sequences of the optic nerve sheath without guidance. This data suggest that unskilled operators are able to achieve the images needed for AI interpretation. However, we also show that image quality differences between ultrasound probes may influence manual ONSD measurements.

## Background

Ultrasonography of the optic nerve sheath (ONS) and measurement of its diameter (ONSD) is a non-invasive technique for detecting elevated intracranial pressure (ICP) (e.g., in traumatic brain injury (TBI) patients) [1–5]. The ONS is a continuation of the dura mater and distends according to cerebrospinal fluid pressure. The technique relies on two key points: (1) An optimal view of the ONS being identified in B-mode ultrasound, (2) an ONSD measurement made 3 mm from the papilla and perpendicular to the orientation of the optic nerve (ON). Ideally, ONSD values greater than a predetermined threshold are suggestive of elevated ICP. For safety, it is important that the ultrasound settings used during ONSD measurement meet the stringent Food and Drug Administration (FDA) limits on acoustic output for ophthalmic ultrasound [1].

Unfortunately, application of the ONSD technique has been mired in inconsistencies in the measurement protocols (resulting in differences in the predictive power) and thresholds across studies [2, 4–10]. Many hypotheses have been suggested for these inconsistencies, including: whether left/right eye measurements are averaged, which orientation (sagittal or transverse) the measurement is made, patient demographics, and whether the ONS itself is being measured. The latter has been the focus of recent scrutiny, as color doppler has shown that acoustic shadow artifacts can create ONS mimics that do not correspond to known anatomy [1, 11]. This has led to the recent proposal of a standard measurement technique via the CLOSED protocol in which the ONS is distinguished from acoustic artifact by color doppler of optic vasculature landmarks [1, 12]. However, as with many of the previous measurement proposals, multi-center clinical trials that assess inter- and intra-operator variability are still needed.

In addition to the standardization of technique, automated ONSD measurement approaches have been proposed to remove the subjectivity and human error in determining the boundaries of the ONS [13–15]. In particular, Moore et al. developed an automated algorithm that could correctly identify and measure ONSD from blind (no B-mode shown to the probe operator) scans of an ocular phantom [14]. This type of AI-aided system, paired with inexpensive, portable, point-of-care ultrasound (POCUS) devices, could allow for diagnosis of elevated ICP at the point-of-injury by reducing the training burden of far forward operators. Such a capability could have an important impact on combat casualty care, mass casualty triage, and triage in low resource environments for traumatic brain injuries.

Here, we evaluate five commercially available POCUS devices for use in ONSD measurement. We present results of a clinician preference survey evaluating the devices for “Image Quality”, “Ease of Acquisition”, “Tactile Feel”, “Software”, and “Overall ONSD” (using a newly designed ocular head phantom). We compare the survey results to a calibration phantom analysis of three different image quality metrics: signal-to-noise (SNR), generalized contrast-to-noise ratio (GCNR), and point-spread function (PSF). Finally, we evaluate the POCUS devices for use in a hypothetical far forward scenario. Operators without previous sonography experience were briefly trained in the concept of ONSD measurement and taught a blind (no visible B-mode) scan procedure to capture the ONS. A week later the unskilled operators performed the procedure using written instructions and ocular head phantoms. The resulting video was scored by how often the ONS was captured and whether the image quality differences between POCUS devices affected ONSD measurement. These studies indicate the potential and considerations for automated ONSD measurement using POCUS devices.

## Methods

### Ultrasound devices and settings

We selected five POCUS devices that were commercially available, vary in transducer technology, form factor, and would be suitable for far forward deployment of ultrasound. Additionally, for the clinician preference study, a cart-based Zonare Z.one SmartCart (with an L10-5 probe) from the intensive care unit (ICU) was used. The Zonare Z.one was chosen as an expensive, cart-based device as a contrast to the less expensive portable POCUS devices. The US devices used are listed in Table 1.

**Table 1:** Characteristics of evaluated US devices

Manufacturer	Model	SDK available?	Transducer type	FDA for optical?	OS	Field of view width	Frequency	Display (size, type, resolution)
Butterfly	Iq	No	CMUT	Y	iOS/Android	33 mm	1–10 MHz	12.4” AMOLED 1752 × 2800
Clarius	L7HD	Yes	Linear	Y	Android	39 mm	4–13 MHz	12.4” AMOLED 1752 × 2800
Sonivate	SonicEye	Yes	Linear^a^	N	Windows	19 mm	5–13 MHz	8” IPS 800 × 280
Interson	SP-L01	Yes	Linear	N	Windows	30 mm	5–10 MHz	15.6” Ultrasharp UHD 3840 × 2160
Sonoque	L5C	No	Linear	N	iOS	38 mm	7.5–10 MHz	11” Retina 2388 × 668
Zonare	Z.one (L10-5)	No	Linear	Y	Proprietary	38 mm	5–10 MHz	17” LCD 1280 × 1024

Software development kit (SDK) available is whether the vendor allows control of the device or live data streaming by 3rd party software. Field of view width is the width of B-mode images using the studies’ parameters. ^a^The Sonivate probe has an additional phased array transducer but only the linear transducer was used in our study.

Except for the Sonivate SonicEye and the Zonare Z.one, the US devices were not sold with a controlling computer or mobile device. Several different controlling devices were used due to different requirements for operating systems and hardware. The Interson SPL01 was operated by a Dell Precision 5540 laptop, the Clarius L7HD and Butterfly iQ were operated by Samsung S7 + tablets, the Sonoque L5C was operated by an 11″ iPad Pro (2020), the Sonivate SonicEye was operated by its proprietary 8″ ruggedized Windows 10 tablet, and the Zonare Z.one was operated by its proprietary cart.

Display characteristics of each probe are described in Table 1. Of the US devices, only the Clarius L7HD, Butterfly iQ, and Zonare Z.one had FDA-approved ophthalmic settings, which were used in the studies. The Interson SPL01 was set to default settings (power 10, “Soft Tissue” enabled, frequency 7.5 MHz). The Sonoque L5C was set to “SmallParts” settings (focus 20 mm, depth 40 mm, dynamic range 50, frequency 10 MHz). The Sonivate SonicEye is unique in that it is a dual probe device, with a linear transducer and a phased array. The SonicEye was set to a proprietary preset (#7) which activated the linear transducer.

### Head phantom

**Fig. 1:**
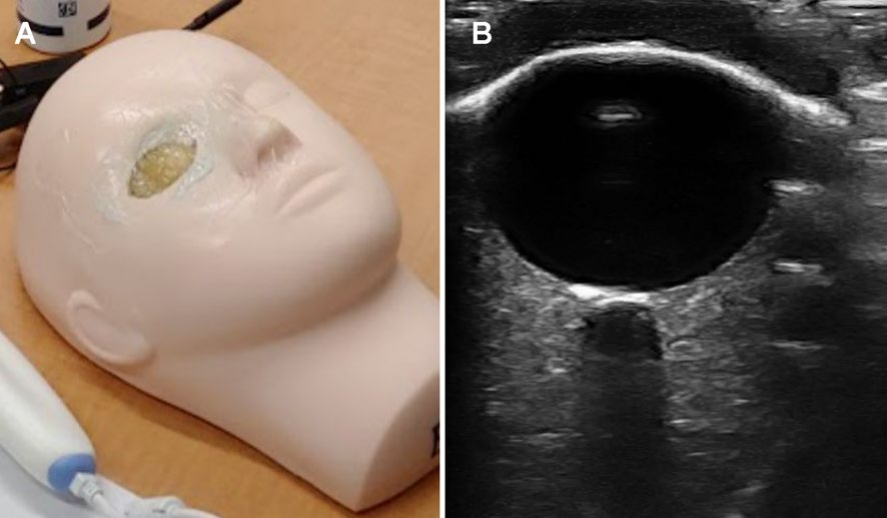
Ocular head phantom. **A**. A picture of the ocular head phantom used in the physician preference and unskilled operator studies. **B**. An example US of the phantom using a Clarius L7HD

The ocular head phantom used in both the physician preference and unskilled operator studies is an in-house design. A detailed protocol for manufacture and bill of materials is available [16]. Briefly, a silicone mannequin head was modified to house a rubber eye socket (molded from an anatomic skeleton). The eye socket contains a gel wax “optic nerve” (ON) suspended in a gelatin mixture. The socket holds a gelatin “ocular orb” and a gelatin mixture eyelid. A picture of the assembled phantom and a representative US is in Fig. 1. The gel wax optic nerve is cast according to different sized, 3D-printed clamshell molds. Due to variability in the final diameter of the optic nerve, the optic nerve width is physically measured along two axes with calipers before final phantom assembly. The hypoechoic “optic nerve” does not contain fine structures such as separate dura mater, subarachnoid space, or a lamina cribrosa. It does provide a circuitous hypoechoic structure with moderately difficult boundaries to measure. We believe that this is an improvement of realism from a previous ONS phantom (housed in a rectangular container) which used a hard plastic disc and its accompanying acoustic shadow to simulate an ONS [14]. We will refer to the gel wax “optic nerve” interchangeably as the optic nerve or the optic nerve sheath for the remainder of the paper.

### Clinician preference study

Clinicians with POCUS experience were recruited from the Surgery Trauma ICU and Neuro ICU to participate in this study. The clinicians were a mixture of advanced practice providers, critical care fellows, and attending physicians. Each participant was given a brief introduction to ocular anatomy and the ONSD measurement procedure by a study coordinator. The coordinator then had the participant evaluate each probe in turn. The order in which participants evaluated the probes followed a Latin Square. For each probe, the coordinator explained to the participant how to use the probe’s software (e.g., zoom, adjust gain, make a ruler measurement). The participant then used the probe to make an ONSD measurement of a head phantom. The study was not designed to evaluate the accuracy of ONSD measurement and therefore the clinician measurements were not analyzed. Note, the Sonivate SonicEye software did not have ruler functionality so participants could only save a still image. After evaluating all six ultrasound probes, each participant then filled out a preference survey [16]. The survey asked the participant to rank the probes (1 being best, 6 being worst) for the following categories: Image Quality, Ease of Acquisition, Software, Tactile Feel, Overall for ONSD.

Analysis-of-variance (ANOVA) was performed individually on each category to determine whether there was a statistically significant difference in rank across probes. We limited post hoc testing to the Butterfly iQ and Clarius L7HD probes due to the relatively small sample size (15) compared to the number of probes (6) evaluated. These two probes were selected as they were the only two POCUS probes with ophthalmic settings and also were evaluated on the same hardware (Samsung S7 + tablet). Wilcoxon signed-rank was used for each category to test the null hypothesis that each probe’s paired ranking came from the same distribution.

### Unskilled operator study

Each participant attended an initial training session followed a week later by the evaluation session. During the training session, participants were described the general anatomy of the ocular orb, the ONS, and the basic principles of ONSD measurement. They were given written instructions for the blind ONSD procedure:

1. Apply ultrasound gel to the eyelid.
2. Place the probe on the center of the eyelid in a horizontal (transverse) orientation
3. Rock the probe to a 30 degree angle upwards
4. Count to eight while slowly moving the probe downwards to an opposing 30 degree angle

After the brief training, each participant evaluated each of the five POCUS probes in turn (the Zonare Z.one was not included in this study due to lack of availability). During the training session only, participants were able to view the B-mode video from each probe’s software (setup by the coordinator) as they performed the blind ONSD procedure on a head phantom. The study coordinator assigned the order each participant evaluated the probes using a Latin Square.

Participants returned a week later for an evaluation session. The participants were given the same written instructions for the blind ONSD procedure. Each participant performed the procedure on three different head phantoms for each probe; each head had a different ONSD (verified by physical caliper measurement on the gel wax ON). The coordinator visually confirmed an appropriate amount of US gel was applied to each phantom. The participant communicated when they were ready to start (i.e., the probe was center on the eyelid at an upward 30 degree angle). The participant would then rock the probe downward and upward until the coordinator recorded approximately 30 s of video using the probe’s system. Unlike the training session, participants could not view the B-mode video during the procedure. The coordinator provided no feedback regarding the quality of the probe position on the phantom. The order in which each participant evaluated the probes followed a Latin Square.

These videos were preprocessed by cropping (removing identifying overlays) and converted to a uniform file format using the open source ITKPOCUS Python package [17]. A single annotator used the *ImageViewer* application to score each video [18]. The annotator was blind to the probe, participant, or phantom corresponding to the video, and videos were served in random order. The annotator counted the number of passes (upward–downward and downward–upward) over the ONS in the video and whether each pass was successful (i.e., whether the entire horizontal span of the ONS was visible in at least one frame during the pass). Passes that failed were due to either the ONS being off-center or occluded by poor transducer contact artifacts. The annotator then picked the clearest frame of the ONS in the video and manually measured the ONSD (3 mm away from the ocular orb and perpendicular to the ONS).

Ordinary Least Squares (OLS) regression was used to estimate the expected difference between the annotator’s manual ONSD measurement and the average caliper measurement of each phantom by device. The Python package, *statsmodels*, was used to calculate the regression model and tests [19].

### Lab analysis

We conducted the lab analyses of the POCUS probes using the CIRS 040GSE calibration phantom [20]. This phantom is different from the head phantoms used in the clinician and unskilled operator studies; it is a commercially available phantom typically used to assess the image quality of US probes. The 040GSE phantom has a combination of precisely located 100 micron diameter wire targets, and 8 mm diameter varying-contrast targets (− 9 db, − 6 db, − 3 db, 3 db, 6 db, and hyperechoic). For each probe and phantom region, triplicate B-mode images were acquired (the probe was removed, repositioned, and the image saved). These images were then manually labeled for each structure in the phantom (e.g., wire, contrast target). The B-mode images and their corresponding label images were then input into our analysis software.

The generalized contrast-to-noise ratio (GCNR) is a measure of overlap between foreground and background pixel intensity probability density functions (PDFs) [21]. Formally, let ${p}_{f}$ and ${p}_{b}$ be the foreground and background PDFs, respectively. The GCNR is defined as$gCNR= 1-\frac{1}{2}{\int }_{-\infty }^{\infty }min\{{p}_{f}(x), {p}_{b}(x)\}dx$. A GCNR of 1, therefore, signifies that an ideal classifier can completely separate the foreground and background pixel intensities (i.e., there is no overlap between PDFs), while a GCNR of 0 means the intensity distributions are identical between foreground and background. A 1.7 mm padded bounding box was computed for each contrast target. The labeled contrast target was the foreground in the GCNR computation and the background was the remaining pixels in the bounding box. The default gain of each device was used for the standard GCNR calculations. For the gain GCNR experiments, the gain settings were adjusted using each device’s software at 0, 20, 40, 60, 80, and 100%.

Signal-to-noise ratio (SNR) was calculated as the mean row-wise intensity divided by the row-wise standard deviation of a 3 mm wide user-specified bounding box right of the vertical wire group. The bounding box contained no structures except for the background gelatin of the phantom.

Point-spread functions (PSF) were estimated from the 100 micron diameter 1, 2, and 3 cm vertical wire targets. A 3 mm line (centered at the centroid of the labeled wire target) was sampled vertically and horizontally. For elevational PSF, the probe was oriented at a 45 degree angle to the wires and the horizontal PSF from the image was recorded. Peaks were identified in each curve using the *find_peaks* method in the *scipy* python package [22]. To average the PSFs from the triplicate images, each image’s PSF was centered at its peak. The resulting centered curves were then resampled and averaged to compute the mean PSF.

The source code and software for the lab analysis is publicly available along with the image dataset [16].

## Results

### Clinician preference study

**Fig. 2:**
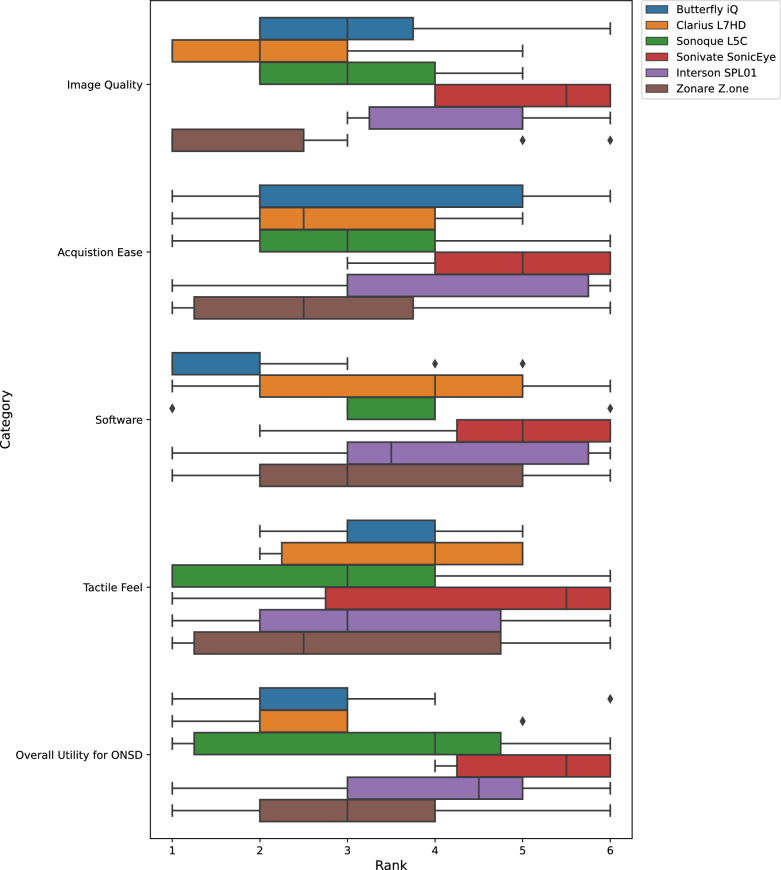
Clinician Preference Survey Ranking Boxplot. Boxplots of clinician rankings (1 is best, 6 is worst) of the POCUS devices by category

Fifteen clinicians from Duke’s Neurological Intensive Care Unit and Surgical Intensive Care Unit attended one of two sessions for the clinician preference study. Each clinician performed ONSD measurement on ocular head phantoms using five POCUS probes and one cart-based clinical probe (Zonare Z.one). Ocular head phantoms varied in ONSD size and were assigned a random order to clinician and probe. Boxplots of clinicians rankings per category are shown in Fig. 2. Mean, median, and standard deviation of ranks scores per probe are shown in Table 2. Note, the Zonare Z.one and Clarius L7HD had similar mean ranks in “Image Quality” (2.1 and 2.3, respectively) and the highest median ranks (1.0 and 2.0). We performed an ANOVA to test the hypothesis that devices had significantly different ranks across the categories: “Image Quality” ($p<0.001$), “Acquisition Ease” ($p=0.014$), “Software” ($p<0.001$), “Tactile Feel” ($p=0.17$), and “Overall Utility for ONSD” ($p<0.001$). Therefore, all categories except for “Tactile Feel” showed a significant difference among POCUS devices. Due to the relatively low sample size ($n=15$) compared to the six devices evaluated, we focused our individual comparisons to the two POCUS devices with ophthalmic settings: the Butterfly iQ and the Clarius L7HD. We used a Wilcoxon signed-rank test (pairwise, nonparametric) to evaluate whether the ranks between the probes. “Image Quality” ($p=0.10$), “Acquisition Ease” ($p=0.64$), “Software” ($p=0.036$), “Tactile Feel” ($p=0.67$), and “Overall Utility for ONSD” ($p=1.0$). Therefore, the Butterfly iQ device had a significantly higher rank for “Software” than the Clarius L7HD. Anecdotally, clinicians positively commented on a feature that provides a zoomed B-mode view of the ruler edge while measuring. 9 out of 15 clinicians had previous experience with the Butterfly iQ and its software, yet 10 had experience with the Zonare Z.one device but its software did not rank as well. None of the participants reported experience with the other POCUS probes.

**Table 2:** Clinician survey ranking of POCUS devices

Probe	Image quality	Acquisition ease	Software	Tactile feel	Overall utility for ONSD
	mean; median (std)				
Butterfly iQ	3.3; 3.0 (1.4)	3.1; 2.0 (1.8)	**2.0; 2.0 (1.2)**	3.4; 3.0 (0.8)	2.7; 3.0 (1.3)
Clarius L7HD	**2.3; 2.0 (1.2)**	**2.8; 2.5 (1.4)**	3.5; 4.0 (1.8)	3.6; 4.0 (1.3)	**2.6; 2.0 (1.5)**
Interson SPL01	4.5; 5.0 (1.2)	3.7; 3.0 (1.9)	3.9; 3.5 (1.6)	3.4; 3.0 (1.8)	4.0; 4.5 (1.7)
Zonare Z.one	**2.1; 1.0 (1.8)**	**2.7; 2.5 (1.6)**	**3.5; 3.0 (1.8)**	**3.0; 2.5 (1.9)**	3.1; 3.0 (1.6)
Sonivate SonicEye	5.1; 5.5 (1.0)	4.8; 5.0 (1.1)	4.9; 5.0 (1.1)	4.5; 5.5 (2.0)	5.2; 5.5 (0.9)
Sonoque L5C	3.2; 3.0 (1.3)	3.5; 3.0 (1.6)	3.5; 4.0 (1.3)	**3.0; 3.0 (1.8)**	3.3; 4.0 (1.8)

Ranks range from 1 (best) to 6 (worst). Best mean rank scores for each category are bolded

### Unskilled operator study

**Fig. 3:**
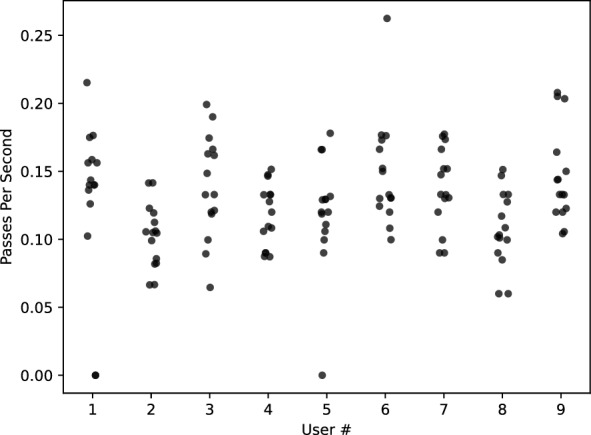
Unskilled Operator Passes per Second. Each point is the number of passes (downward-upward, upward-downward) across the eye per individual video. The unskilled operators were instructed to count to eight, or 0.125 passes per second

**Fig. 4:**
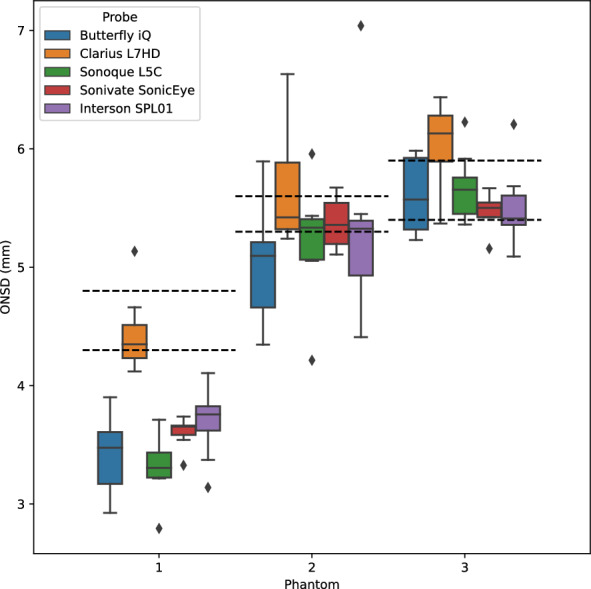
Manual ONSD Measurement of Unskilled Operator Video Boxplot. A boxplot of manual ONSD measurement of unskilled operator video by a single annotator. The dotted lines represent minimum and maximum physical caliper measurements of the gel wax ONS prior to phantom assembly

Nine adults without previous experience in ultrasound participated in the unskilled operator study. The study resulted in 135 (9 participants, 5 probes, 3 phantoms) B-mode videos (approximately 30 s each). The videos were manually annotated for number of passes over the ONS, number of passes that had at least one frame with the ONS in view, and a manual ONSD measurement. The number of passes per second of video is shown in Fig. 3. The percentage of successful passes is shown in Table 3. A boxplot of manual ONSD measurements on the unskilled operator video is shown in Fig. 4. Model fit parameters are shown in Table 4. Note, all probes aside from the Clarius L7HD have a statistically significant negative coefficient, denoting an underestimate of ONSD.

**Table 3:** Pass rate for unskilled operator video

Device	Pass success rate	
	Mean	Std
Butterfly iQ	0.921869	0.186915
Clarius L7HD	0.917308	0.163672
Interson SPL01	0.832378	0.309757
Sonivate SonicEye	0.516728	0.350693
Sonoque L5C	0.923077	0.250188

**Table 4:** OLS regression for manual ONSD measurement

Probe	Coefficient	Std error	t	P >|t|	[0.025	0.975]
Butterfly iQ	− 0.5203	0.112	− 4.656	0	− 0.741	− 0.3
Clarius L7HD	0.1415	0.114	1.242	0.216	− 0.084	0.367
Interson SPL01	− 0.358	0.104	− 3.432	0.001	− 0.564	− 0.152
Sonivate SonicEye	− 0.4586	0.124	− 3.704	0	− 0.703	− 0.214
Sonoque L5C	− 0.5604	0.083	− 6.755	0	− 0.724	− 0.396

### Lab analysis

**Fig. 5:**
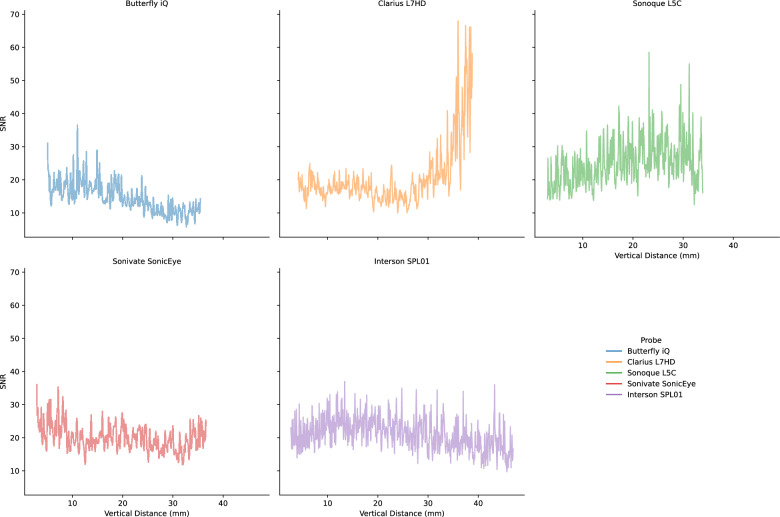
SNR over Vertical Distance. SNR curves per device by vertical distance from the transducer

**Fig. 6:**
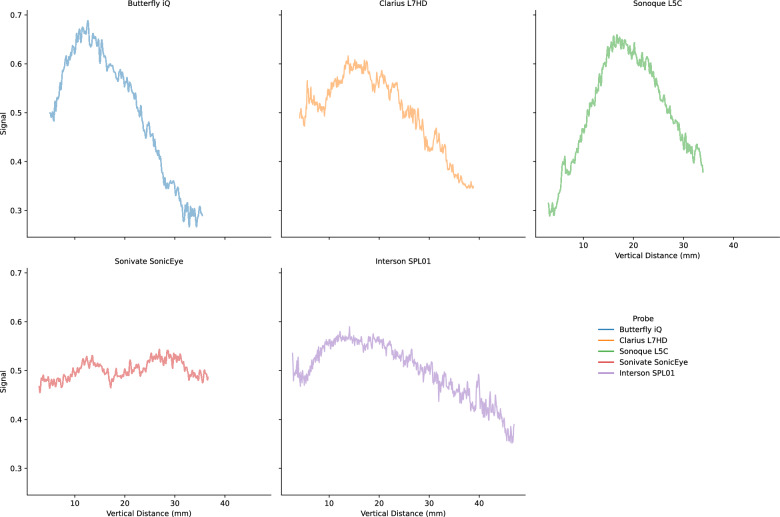
Signal over Vertical Distance. Signal (intensity) curves per device by vertical distance from the transducer

**Fig. 7:**
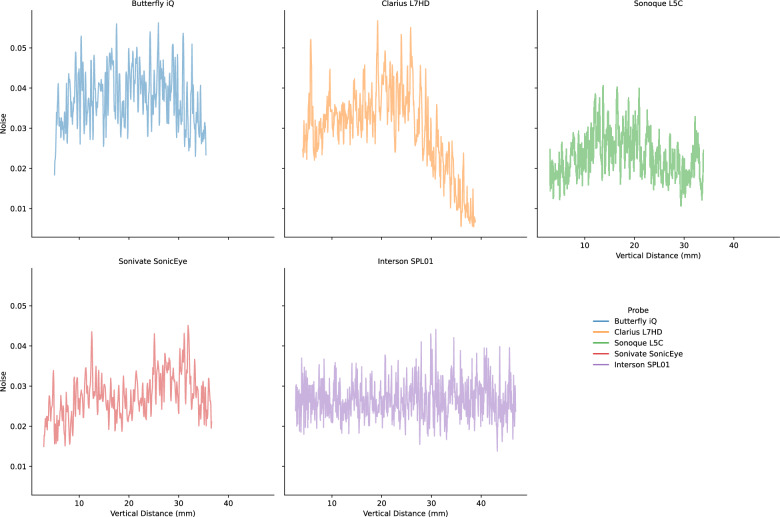
Noise over Vertical Distance. Noise (standard deviation of intensity) curves per device by vertical distance from the transducer

**Fig. 8:**
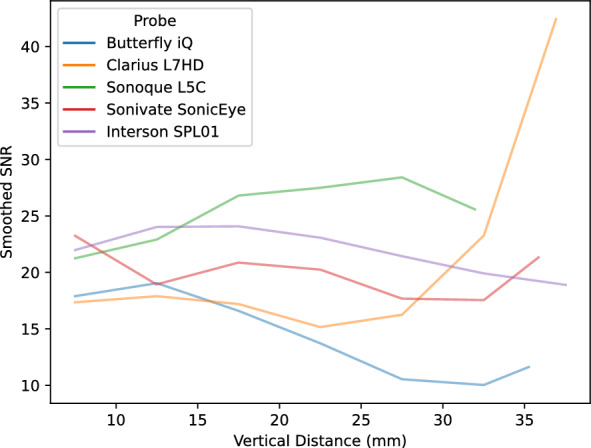
Mean Window SNR over Vertical Distance. Individual SNR curves were smoothed by averaging by a 5 mm window at 5 mm intervals. Vertical distance is the depth of the measurement from the transducer

**Fig. 9:**
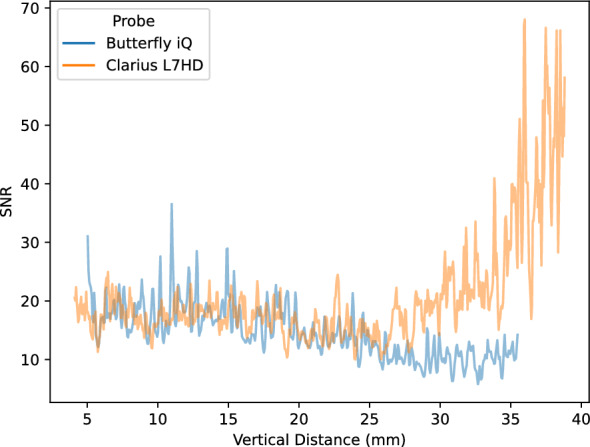
Clarius L7HD vs Butterfly iQ SNR over Vertical Distance

**Fig. 10:**
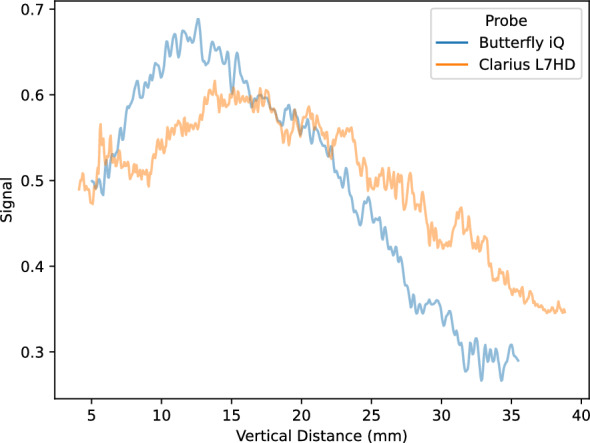
Clarius L7HD vs Butterfly iQ Signal over Vertical Distance

**Fig. 11:**
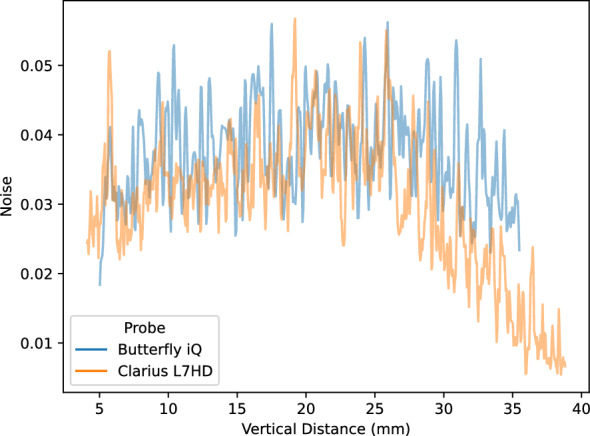
Clarius L7HD vs Butterfly iQ Noise over Vertical Distance

Lab analyses were conducted on the five POCUS devices. The CIRS 040GSE calibration phantom was used. For each structure used in measurement (wire or contrast targets), three B-mode images were acquired (the probe was removed and repositioned between images). SNR ratios by vertical depth are shown in Fig. 5. SNR values can be driven by changes in signal (Fig. 6) and noise (Fig. 7). As the SNR curves themselves are noisy (even though they are averaged over three images), we additionally computed window-averaged curves (Fig. 8). We plotted direct comparisons of only the Clarius L7HD and Butterfly iQ. These were the only devices with FDA-approved ophthalmic settings (Figs. 9, 10, 11). SNR values between the Clarius and Butterfly devices are similar until they diverge at 25 mm. The higher Clarius SNR is driven by a slower drop-off in signal and decrease in noise.

**Fig. 12:**
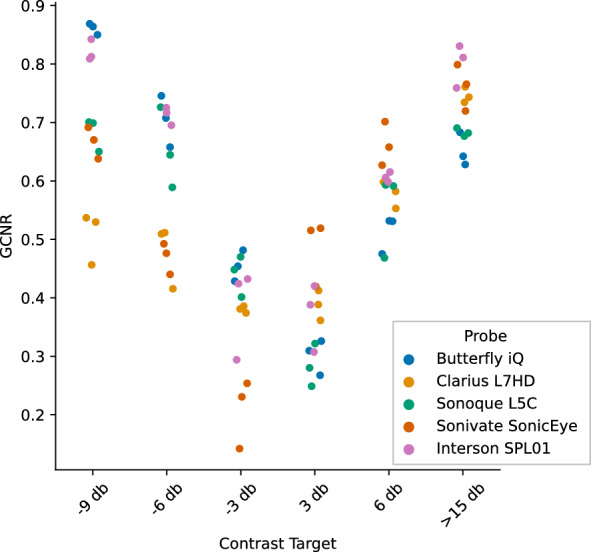
GCNR across Contrast Targets by Device. Each point is a measurement from 1 of 3 replicate images per contrast target and device

**Fig. 13:**
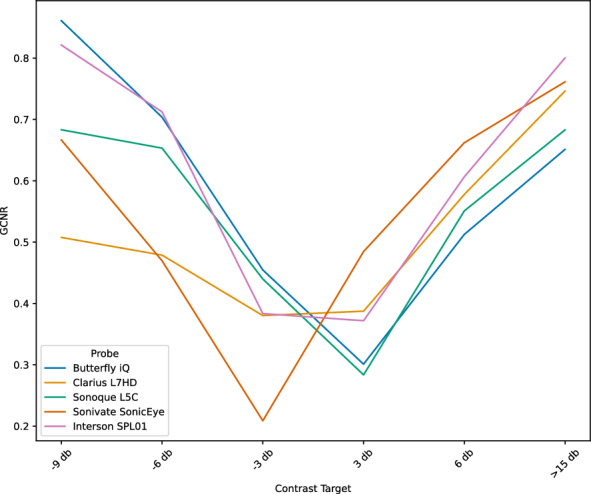
Mean GCNR across Contrast Targets by Device. The mean GCNR values across replicates is plotted per device across contrast targets

**Fig. 14:**
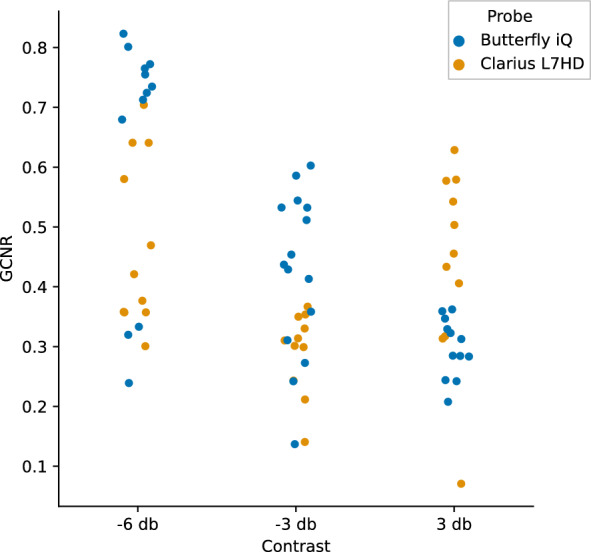
GCNR Butterfly iQ and Clarius L7HD across Contrast Targets and Gain. Each point is a measurement from 1 of 3 replicate images and a given gain setting. The highest points per device and contrast target should represent optimal gain settings

**Fig. 15:**
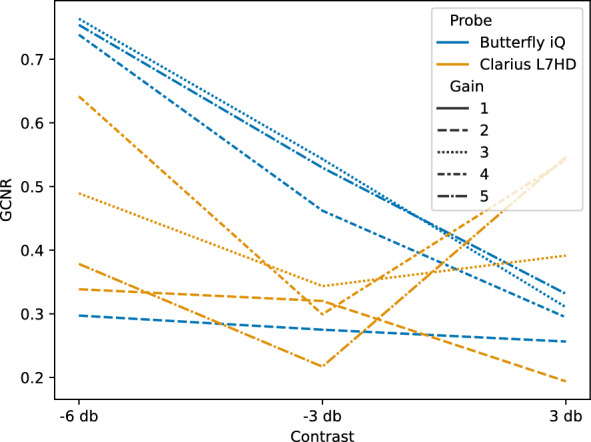
Mean GCNR Butterfly iQ and Clarius L7HD across Contrast Targets and Gain. Each line is the mean GCNR value across triplicate images and represents the performance of an individual gain setting across contrast targets. Note, the line corresponding to the lowest gain setting (Gain 1) is not visible as the contrast targets were not distinguishable from background for both devices

GCNR is a measure of intensity difference between foreground objects and background. Figures 12, 13 show the individual and mean GCNR values per device and contrast target (using default gain settings). The U-shaped trend seen across devices is expected, as the center contrast targets are most similar to the background medium. Gain settings can directly affect this measure; too low of a setting will make the darkest contrast targets indistinguishable from background and too high of a setting will saturate the hyperechoic targets. We varied the gain settings (0, 25%, 50%, 75%, 100%) on the two ophthalmic-supporting devices (Clarius L7HD and Butterfly iQ). A scatterplot showing GCNR values from individual triplicate images across all gain settings is shown in Fig. 14. The Butterfly iQ has the highest GCNR values for the -6 and -3 db contrast targets on the optimal gain settings, while the Clarius L7HD outperforms on the 3 db target. The effect of individual gain settings is shown in Fig. 15.

**Fig. 16:**
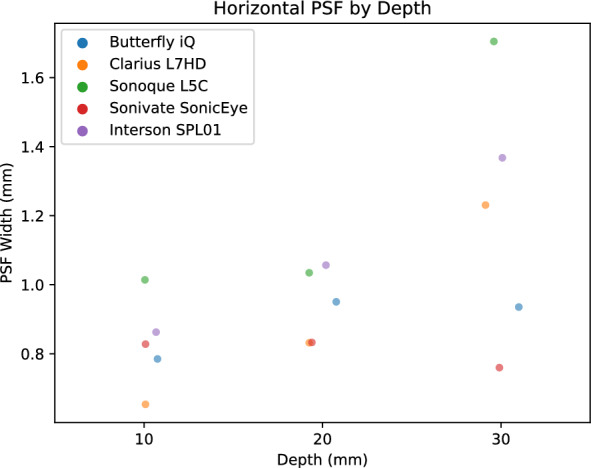
Mean Horizontal PSF by Device and Depth. Each point represents the mean PSF width in the horizontal direction of the vertical wire target at that depth

**Fig. 17:**
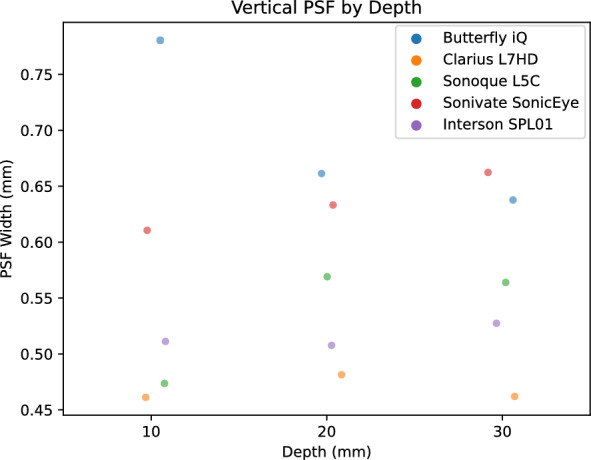
Mean Vertical PSF by Device and Depth. Each point represents the mean PSF width in the vertical direction of the vertical wire target at that depth

**Fig. 18:**
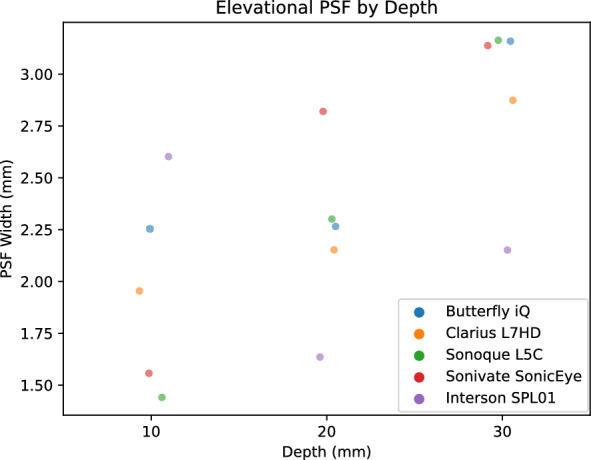
Mean Elevational PSF by Device and Depth. Each point represents the mean PSF width in the elevational direction of the vertical wire target at that depth

**Fig. 19:**
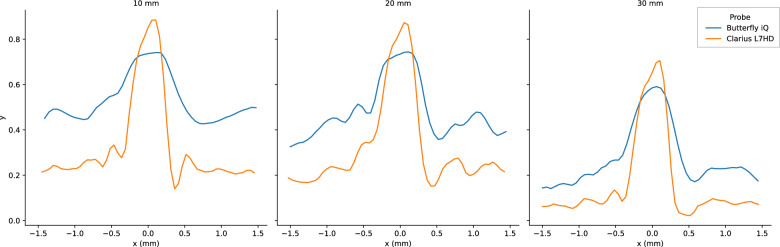
Butterfly iQ and Clarius L7HD Vertical PSF. Mean vertical PSF curves across vertical depth

**Fig. 20:**
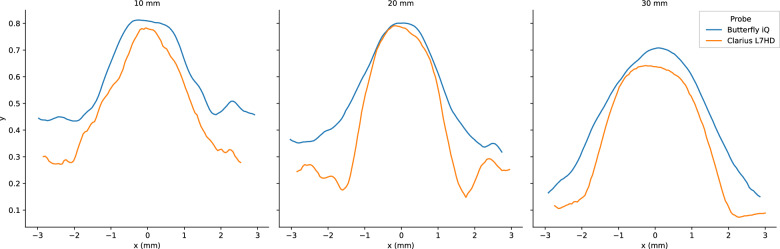
Butterfly iQ and Clarius L7HD Elevational PSF. Mean elevational PSF curves across vertical depth

**Fig. 21:**
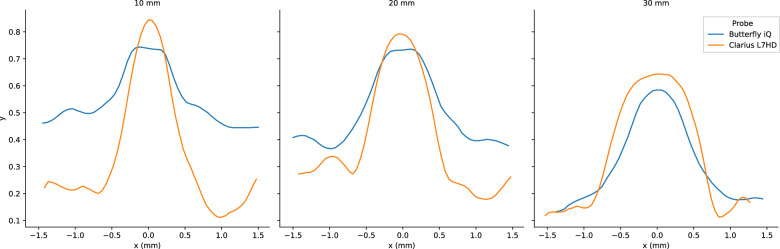
Butterfly iQ and Clarius L7HD Horizontal PSF. Mean horizontal PSF curves across vertical depth

PSF width is a measure of the blurring and effective resolution of an US device. We calculated PSF curves from each of three 100 micron diameter wire targets at 1, 2, and 3 cm. We measured the PSF width in the horizontal (Fig. 16), vertical (Fig. 17), and elevational (Fig. 18) directions for each US device. Each of the devices had varying performance across the direction. We attribute this to the different number of transducer elements, signal processing, and focal depth of the devices. Again, we reserved direct comparison for the two ophthalmic probes. The Clarius L7HD had superior PSF width values over the Butterfly iQ in the vertical (Fig. 19) and elevational (Figure 20) directions across all targets. The L7HD also had better values in the horizontal (Fig. 21) direction at 1 cm and 2 cm, but was surpassed by the Butterfly iQ at 3 cm.

## Discussion

**Fig. 22:**
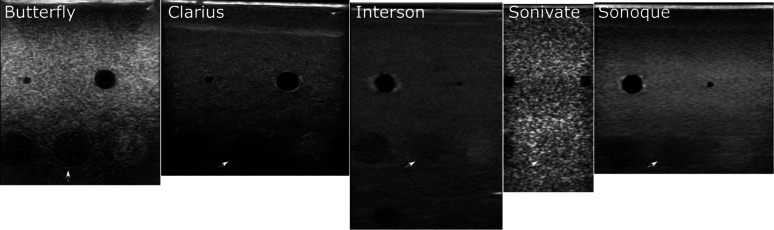
Varying Image Quality across POCUS Devices. Images of the same phantom structure (-3 db target, white arrow) highlight qualitative differences in image quality across POCUS devices

Low-cost, portable, ultrasound could allow for ONSD measurement to occur far-forward to the point-of-injury. We sought to evaluate the differences in commercially available POCUS devices with respect to their potential use in a future automated ONSD measurement system. At first glance, there are obvious qualitative differences between images of the same object. The calibration phantom in Fig. 22 is a perfect example. Each device has differences in transducer hardware, frequency ranges, and signal processing filters. We see that these differences affect the clinician preference for the devices; 4 out of 5 tested categories in our clinician study showed statistically significant differences across devices.

The clinician study alone, however, has limitations. Anecdotally, the different display hardware seemed to impact preference, though this was controlled between the two FDA-approved ophthalmic POCU devices (Clarius L7HD and Butterfly iQ). Additionally, the Zonare Z.one and the Interson SPL01 were controlled by non-touch screen devices, and the remaining probes were controlled by tablets. FDA ophthalmic requirements greatly restrict the power levels which would negatively impact image quality; however, the Clarius L7HD had the highest mean and median rank for “Image Quality” of the POCUS devices (only the reference clinical device, the more expensive, less portable Zonare Z.one, scored higher). In fact, several of the clinicians remarked they were impressed by the image quality of the Clarius L7HD compared to the more expensive Zonare Z.one device. While this study was not blinded or controlled to solely look at quality, it suggests that the utility of POCUS devices are not limited by their portable form factor or low cost.

POCUS manufacturers, such as Clarius and Butterfly, market the mobile phone interface of their ultrasound platform. We note that display size appears to be an important factor with respect to perception of image quality. One clinician commented that the images from the Butterfly iQ seem significantly better on the large, bright, high resolution display of the Samsung S7 + . This contrasts with the lowest rank given to the Sonivate SonicEye, which had the smallest, poorest resolution display, but whose images performed relatively well according to lab analysis metrics. Additionally, the poor ranking given to the SonicEye for “Overall ONSD” is likely driven by the software’s lack of a ruler feature, which prevented the clinicians from making manual ONSD measurement. The display fidelity of the POCUS image seems nearly as important as the ultrasound platform.

For each POCUS device performance varied across the three lab metrics (SNR, GCNR, PSF width). No particular device outperformed others across all metrics. We performed paired comparisons between the Clarius L7HD and Butterfly iQ (the only POCUS devices that had FDA-approved ophthalmic settings). Because the Clarius L7HD had a higher mean ranking (2.3) for “Image Quality” than the Butterfly iQ (3.3), we looked for similar trends across the lab metrics. For SNR, both probes have similar values until they begin to diverge at 2.2 cm, with the Clarius L7HD maintaining higher values. As the expected depth for ONSD measurement is anywhere from 2.4 to 2.9 cm, this divergence may be significant. The L7HD does outperform in all measures of PSF width (vertical, elevational, horizontal) except for the horizontal PSF at 3 cm where the iQ surpasses it. Though the Butterfly iQ may have superior horizontal resolution near where an ONSD measurement may occur, that advantage may be confounded by its inferior elevational, or out-of-plane, resolution. Our contrast measurement, GCNR, also shows competing results, as the Butterfly iQ has superior values for the hypoechoic targets (-6 db, and -3 db) but inferior values for the hyperechoic target (3 db). These trade-offs across the quantitative metrics suggest that a POCUS device’s ultimate performance will be task-specific.

One of the main goals of this study was to evaluate these POCUS devices for use by unskilled (or lightly trained) operators. We believe that this type of operator represents a typical first responder. Given the nuance of manual ONSD measurement, such as determining whether the ONS is clearly in view or occluded by an acoustic artifact, we believe these operators will necessarily be supported by an AI system that not only makes the ONSD measurement but also determines which video frames are appropriate for measurement. We sought to determine whether or not the operators could record video frames of the ONS by placing the probe on the center of the eyelid and rocking it slowly in a transverse orientation. Critically, the operators were unable to see the US video as they performed the scan. If the operators could record the ONS without viewing the live B-mode video, then an AI system that lacks user guidance may be feasible for ONSD measurement.

We found that the operators were able to perform a blind scan of the ONSD with over 90% of passes over the ocular phantom capturing a clear frame of the ONS for a majority of the POCUS devices. Significantly less passes (52%) were successful using the Sonivate SonicEye. This demonstrates a trade-off; the smaller transducer size allows for better maneuverability around the eye, but the relatively small field of view results in the ONS occasionally moving out of frame during the blind scan. This suggests that smaller transducer widths may necessitate some sort of feedback to the user for guidance.

We next investigated whether the noted differences in image quality metrics may result in differences in manual ONSD measurement. We collected the video from the unskilled operators and had a single annotator measure the ONSD in random order. The results show that the Clarius L7HD video resulted in a significantly more accurate ONSD measure, though the effect seems inconsistent with the largest ONS head phantom. The below background dips in PSF curves (see Fig. 21) suggest that the Clarius L7HD has edge-enhancing post-processing, which may lead to more pronounced boundaries of the ONS (especially at smaller diameters). These differences suggest device model may be a contributing factor to inconsistency of ONSD threshold [4].

We note that there are limitations to the operator study. The ocular phantoms used do not capture some of the confounding issues with the human ONS, such as blooming artifacts, ON tortuosity due to Bell’s phenomenon, or patient movement [1]. The definition of a “successful pass” used here (the horizontal span of the ocular phantom’s ONS being within a video frame) does not mean that the operator captured a single video frame that would meet clinical guidelines such as the CLOSED protocol [1]. However, it is likely that an AI system that evaluates an entire sequence of video would not require as stringent guidelines for an individual frame.

This study highlights the differences that exist across commercially available POCUS devices. These differences persist despite manufacturer-provided ophthalmic presets used on two devices. Most importantly, we have shown those differences can affect the ONSD measurement itself. The ocular model was accurate and uniformly created to allow repeated high fidelity POCUS assessments. The model is more simple than the human eye. Specifically, the model lacks the lamina cribrosa which is thought to cause a shadow artifact. This leads us to hypothesize that these differences may be even more apparent in human ONSD measurement. We suggest care be taken when procuring new US devices as this may affect the ONSD threshold for elevated intracranial pressure. We plan to further investigate the differences between the Clarius L7HD and Butterfly iQ in our ongoing clinical study that involves automated AI interpretation of these images (clinicaltrials.gov, NCT04515212).

## Conclusions

We show a statistically significant difference in manual ONSD measure between two POCUS devices with manufacturer-provided ophthalmic presets. We summarize differences in image quality between five POCUS devices across three measures in a calibration phantom analysis. Finally, we show that operators with no previous US experience were able to capture the ONS in frame, without viewing B-mode, during a head phantom study. This result suggests that future automated ONSD AI systems may allow first responders without ultrasound expertise to make the measure, a capability that would facilitate rapid evacuation and improved survival from ICP. An ongoing human study (clinicaltrials.gov, NCT04515212) will investigate the differences between the FDA-approved ophthalmic POCUS devices in manual ONSD measurement and those from an automated AI system.

## Data Availability

The datasets used and/or analyzed during the current study are available from the corresponding author on reasonable request. The statistical analyses and image quality analyses are available at the 2023-Eval-POCUS-ONSD repository, https://github.com/KitwareMedicalPublications/2023-Eval-POCUS-ONSD.

## Abbreviations

ONSD: Optic nerve sheath diameter
ONS: Optic nerve sheath
ON: Optic nerve
US: Ultrasound
POCUS: Point-of-care ultrasound
PSF: Point-spread function
US: Ultrasound
TBI: Traumatic brain injury
GCNR: Generalized contrast-to-noise ratio
SNR: Signal-to-noise ratio
ICP: Intracranial pressure
AI: Artificial intelligence
PDF: Probability density function
